# Hemophagocytosis of the Hilar Pulmonary Lymph Nodes Is a More Sensitive Indicator of the Severity of COVID-19 Disease than Bone Marrow Hemophagocytosis

**DOI:** 10.3390/diseases12100241

**Published:** 2024-10-03

**Authors:** Amira Jusovic-Stocanin, Elke Kaemmerer, Hannah Ihle, Angelina Autsch, Sandra Kleemann, Juliane Sanft, Michael Hubig, Gita Mall, Nikolaus Gassler

**Affiliations:** 1 Section of Pathology, Institute of Forensic Medicine, Jena University Hospital, 07747 Jena, Germany; 2 Department of Pediatrics, Jena University Hospital, 07747 Jena, Germany; 3 Institute of Forensic Medicine, Jena University Hospital, 07747 Jena, Germany

**Keywords:** autopsy, bone marrow, COVID-19, hemophagocytic lymphohistiocytosis, lymph nodes

## Abstract

**Simple Summary:**

A severe, fatal COVID-19 disease is often associated with the occurrence of hemophagocytosis. Hemophagocytosis is particularly pronounced in the hilar lymph nodes, indicating compartmentalized human host responses to life-threatening infections.

**Abstract:**

In systemic hyper-inflammation, as in severe COVID-19 disease, there are pronounced disorders of the hematological and lymphatic systems with prognostically relevant hemophagocytosis of the bone marrow. The current work aimed to address the importance of hemophagocytosis in the lymph nodes of patients with severe COVID-19 disease. From 28 patients who died of severe COVID-19 infection, samples of the vertebral bone marrow and lymph nodes from the cervical, hilar, para-aortic, mesenteric and inguinal locations were morphologically and immunohistologically (CD163, CD68, CD61, CD71, CD3, CD20, CD138) examined for the possible presence of hemophagocytosis. In the single-center study at the University Hospital Jena, a total of 191 hemophagocytes were found in the bone marrow and a total of 780 hemophagocytes in the lymph nodes in a standardized area of 21,924 mm^2^ per tissue sample. With 370 hemophagocytes, hilar lymph nodes were most frequently affected (370/780; 47.44%; 95%-CI: [43.94, 50.95]), followed by cervical lymph nodes (206/780; 26.41%; 95%-CI: [23.41, 29.59]), para-aortic lymph nodes (125/780; 16.03%; 95%-CI: [13.58, 18.73]) and inguinal/mesenteric lymph nodes (79/780; 10.13%; 95%-CI: [8.155, 12.4]). Based on the standard area (21,924 mm^2^), the difference in the number of hemophagocytes in the bone marrow and in the hilar lymph nodes was statistically significant (*p* < 0.05), while this did not apply to the lymph nodes from the other locations. In fatal COVID-19 disease, hemophagocytosis is particularly found in the hilar lymph nodes and is therefore a better indicator of the severity of the disease than hemophagocytosis in the bone marrow. The findings provide some evidence for the concept of compartmentalized human host responses to life-threatening infections.

## 1. Background

Patients with severe and fatal COVID-19-associated pneumonia may show disproportionately high signs of systemic hyper-inflammation with cytokine storm, also known as hemophagocytic lymphohistiocytosis (HLH) [1,2,3,4]. In principle, HLH is a hyper-inflammatory condition divided into a primary (familial) or secondary variant (sHLH) when associated with triggers including viral infections, certain chemotherapies or malignancies [5]. The molecular mechanisms behind sHLH are in evaluation and discussion in order to identify therapeutic targets [6,7]. The clinical features of HLH are prolonged fever, hepatosplenomegaly, hypertriglyceridemia, cytopenia, liver damage, jaundice, elevated ferritin levels and central nervous system involvement [8].

The hyper-inflammation response to SARS-CoV-2 in sHLH is suggested as a pathogenic inflammatory process that stimulates and accompanies the development and progression of acute respiratory distress syndrome (ARDS), as well as the systemic manifestation of severe and fatal COVID-19 disease. In addition to SARS-CoV-2, a variety of infectious agents are known as sHLH triggers, including EBV, CMV, tick-borne infections, fungi and protozoa [9]. In SARS-CoV-2 infection, the occurrence of sHLH with nucleated or non-nucleated hemophagocytosis in the bone marrow was suggested as an indicator for a worse prognosis [10,11].

In contrast to a plethora of clinical signs and criteria that are indicative of sHLH, hemophagocytosis is the only microscopical criterion indicative of sHLH. There is some evidence that non-nucleated erythrophagocytosis alone is a non-specific finding, while hemophagocytosis of nucleated cells is strongly associated with HLH [12]. However, the finding of strong erythrophagocytosis in a specific clinical context, like in an infectious disease such as COVID-19, should encourage consideration of sHLH. In such cases, the histiocytes can be very prominent, with a sack-like appearance caused by the plethora of ingested erythrocytes within the cytoplasm of the histiocytic cell [13].

The course of COVID-19 is influenced by host factors such as lymphocytopenia and the occurrence of the so-called ‘cytokine storm, where serum levels of TNF-α, IL-6 and IL-10 are negatively correlated with the amount of T cells [14,15]. In the pathogenesis, monocytes and macrophages containing SARS-CoV-2 virus particles expressing IL-6 are probably involved. The presence of IL-6 + macrophages was associated with severe depletion of lymphocytes from the spleen and lymph nodes. Viral SARS-CoV-2 RNA has been detected in the stromal and immune cells of para-aortic, cervical, hilar and mesenteric lymph nodes [16].

In SARS-CoV-2, the histomorphology of lymph nodes is highly variable. The patterns are dominated by a decrease in lymphocytes with absent germinal centers associated with sinus and vascular dilatation, an increase in reactive plasmablasts, sinus histiocytosis with focal hemophagocytosis, thrombo-inflammatory effects, and occasional cystic transformation with stroma fibrosis and accumulation of siderophages [17,18]. Histological features such as edema and activation of plasmablasts are visible in the lymph nodes of early fatalities, while macrophage activation and a subtle germinal center response are dominant further in the course of disease and in more severe infection.

In a previous study from our group, hemophagocytosis in the bone marrow, but not in lymphatic nodes, of deceased individuals with severe COVID-19, was investigated and suggested as a relevant indicator of a fatal course of the infectious disease [10]. In the present study, lymphatic nodes of several anatomical regions of COVID-19 patients were investigated for hemophagocytosis to provide proof of locoregional differences.

## 2. Materials and Methods

### 2.1. Autoptic Tissues

Tissues from 28 autopsies carried out between July 11 and 19 December 2020 at the University Hospital Jena were included in the study. All patients (*n* = 28) had died of a severe COVID-19 infection and were positive for SARS-CoV-2 before death using a clinical diagnostic PCR test. The median time between death and autopsy was about 5 h. All tissues were obtained according to current guidelines for tissue preservation at autopsies. In accordance with these specifications, cervical, hilar, para-aortic, mesenteric and inguinal lymph nodes as well as vertebral bone marrow from the Th10 to L3 location were preserved for the study. The local Ethics Committee of the University Hospital Jena provided a positive vote to carry out the study (registration no.2020-1773, 5 May 2020).

### 2.2. Processing of Lymph Nodes and Bone Marrow

The autopsy-obtained tissues were routinely processed as formalin-fixed and paraffin-embedded (FFPE) samples. The bone marrow samples were decalcified using EDTA-formalin. Approximately 2 μm thick tissue sections were stained with the standard hematoxylin–eosin and Prussian blue stains. Immunostaining was carried out to reliably identify hemophagocytosis and to specifically display cell types. The following antibodies were used to image monocytes and macrophages: anti-CD163 (Novocastra, Newcastle, UK; NCL-CD163) and anti-CD68 (Dako, Glostrup, Denmark; M0876). To identify the cells of hematopoiesis, the following were used: anti-CD61, Dako, M0753 (megakaryocytes/platelets); anti-CD71, Zytomed, Berlin, Germany, ACI 3110 B (erythroid cells); and anti-myeloperoxidase, Dako, IR511 (myeloid cells). To ensure the allocation of lymphatic cells, immunostaining was carried out with anti-CD3 (Dako, IR503), anti-CD20 (Dako, IR604) and anti-CD138 (Dako, IR642). All immunostainings were performed using routine protocols that run on the Omnis machine (Agilent, Santa Clara, CA, USA). All immunostainings were confirmed by controls carried out in parallel.

### 2.3. Identification and Counting of Phagocytes

Tissue slides were microscopically examined at low magnification using the Zeiss Axioscope 506 (Jena, Germany). Regions of interest (ROI) were defined, including different histological areas of the examined tissue. In the lymph node, the marginal sinus and the subcapsular niche, as well as areas of the interfollicular zone, were randomly selected. In the bone marrow, the spongiosa cavities were preferentially examined, but the cortical and paracortical areas were spared. According to these criteria, the areas of the tissues were randomly selected. Each individual area had an expanse of 312.55 × 250.61 µm. Ten such areas were selected per tissue, so that the ROI per individual tissue was 0.783 mm^2^. The areas were documented at 400× magnification using a Zeiss Axioscope 506 (zen 2.6 software, blue edition, Jena, Germany). The histomorphological images of the ROI were evaluated for hemophagocytosis independently by two morphologists (AJ and NG) using the open-source platform Fiji for biological image analysis.

### 2.4. Medical Records

Data that demonstrated the severity of COVID-19 infection as well as other clinical data were specifically taken from the patient files. The data are summarized in Table 1.

### 2.5. HLH Score Calculation

The HLH score was calculated as described previously [10]. Where available, the last laboratory data before the patient’s death were used, as well as the respective organ weights in relation to body weight, to assess hepatomegaly and splenomegaly. For calculation, the following HLH algorithm was used: https://saintantoine.aphp.fr/score/, accessed on 11 September 2024.

### 2.6. Statistical Analysis

Data were addressed with percentages for nominal values. For ratio values, the median and range were determined. The *t*-test (one- and two-tailed), as well as the MWU test, was applicable to compare the means of two groups with normal distribution (Kolmogorov–Smirnov) and homogeneous variances. The IBM-SPSS was used for procedures. A *p*-value of <0.05 was assumed to be statistically significant. Confidence limits for proportions were computed using mid-p exact calculation [https://www.openepi.com/Proportion/Proportion.htm, accessed on 23 September 2024.

## 3. Results

### 3.1. Clinical Patient Characteristics

In the single-center study, 28 patients with a severe and fatal course of COVID-19 were included [19 (68%) males; 9 (32%) females]. The median age of all patients was 80 years (range 52–87 years), with a median age of 78 years for men and 83 years for women.

If a BMI more than 29.9 is defined as severely overweight, 25 patients (25/28; 89.29%; 95%-CI: [73.55, 97.2]) were affected [18 men (all patients: 18/28; 64.29%; 95%-CI: [45.52, 80.23]; all men: 18/19; 94.74%; 95%-CI: [76.67, 99.74]); 7 women (all patients: 7/28; 25%; 95%-CI: [11.64, 43.3]; all women: 7/9; 77.78%; 95%-CI: [43.79, 96.09])]. Based on an ideal BMI of 24–29 at an age of over 65 years, only 13 patients had an increased BMI (13/28; 46.43%; 95%-CI: [28.77, 64.79]) [10 men (total 10/28; 35.71%; 95%-CI: [19.77, 54.48]) and 3 women (total 3/28; 10.71%; 95%-CI: [2.799, 26.45])].

All patients (*n* = 28) had been hospitalized as a result of COVID-19 disease. With regard to mechanical ventilation due to COVID-19 disease, evaluable data from 24 patients were available (24/28; 85.71%; 95%-CI: [69.05, 95.29]). Based on this cohort (*n* = 24), the majority of patients were ventilated (16/24; 66.67%; 95%-CI: [46.36, 83.16]). Five patients were ventilated for a maximum of 7 days (5/24; 20.83%; 95%-CI: [8.059, 40.3]), while eleven patients (11/24; 45.83%; 95%-CI: [26.96, 65.66]) were ventilated for longer than 7 days (maximum 40 days, some with ECMO).

In the overall cohort (*n* = 28), the majority of patients showed comorbidities (25/28; 89.29%; 95%-CI: [73.55, 97.2]). The number of patients who had more than one additional disease was dominant (23/25; 92%; 95%-CI: [76, 98.64]; total 23/28; 82.14%; 95%-CI: [64.76, 93.15]). All patients with only one comorbidity were tumor patients (3/25; 12%; 95%-CI: [3.145, 29.28]; total 3/28; 10.71%; 95%-CI: [2.799, 26.45]). Important clinical data for the 28 patients are summarized in Table 1.

### 3.2. Hemophagocytosis Is Found in the Lymph Nodes of Patients Who Have Died from Severe COVID-19

**Figure 1:**
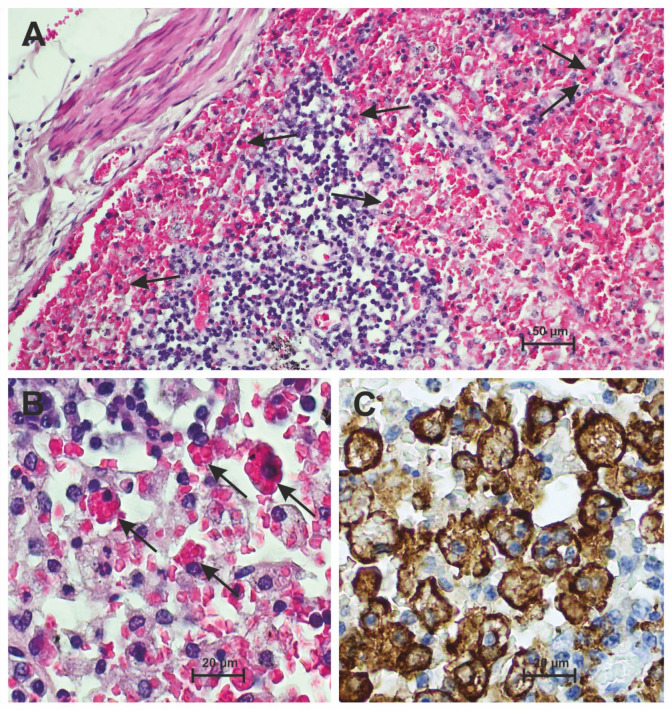
Subcapsular sinus and adjacent parenchyma of a hilar lymph node in severe COVID-19 disease. (**A**) Architecturally disturbed lymph node with hemorrhage of the sinus and numerous hemophagocytes with erythrophagocytosis. Examples are marked with arrows. At the bottom of the picture there is sparse anthracotic pigment (H&E). (**B**) Higher magnification of non-nucleated hemophagocytosis (arrows) of the lymph node (H&E). (**C**) Numerous hemophagocytes in the anti-CD163 immunostaining, some of them confluent.

Lymph nodes in the cervical, hilar, para-aortic, mesenteric and inguinal locations were histologically examined. The lymph nodes generally showed age-dependent changes to varying degrees. In particular, the inguinal lymph nodes showed a lipomatous substitution of the lymphatic tissue, while the hilar lymph nodes were frequently anthracotic and fibrosed. Hyalinosis and small dystrophic calcifications were occasionally present. Neoplastic infiltrates were found in the lymph nodes of two patients (2/28; 7.14%; 95%-CI: [1.215, 21.65]), each of which could be assigned previously known malignancies (chronic lymphatic leukemia and carcinoma). In addition to the changes mentioned, the lymph nodes showed variable histological findings that could be causally linked to COVID-19 infection. Histological findings were dominated by reduced lymphatic cellularity with lymphatic depletion and loss of the germinal center reaction. This was often accompanied by plasmacytosis and sinus ectasia with enlarged histiocytic cells, accumulation of macrophages, and hemorrhage of the tissue. As an expression of an excessive immune response in the case of sHLH or macrophage activation syndrome, indicators of hemophagocytosis, in particular non-nucleated erythrophagocytosis, were repeatedly seen in the lymphatic tissue. These findings were particularly frequent and pronounced in the paracortical zone and subcapsular sinus of the lymph nodes. In addition, such lymph nodes often showed fresh bleeding and the circumscribed retention of siderophages. Histological essentials are illustrated in Figure 1.

Non-nucleated erythrophagocytosis was found histologically and structurally in lymph nodes from all locations examined, but the number of erythrophagocytes was significantly variable and nucleated hemophagocytosis in lymph nodes was not detectable. A total of 780 non-nucleated erythrophagocytes were found in all of the examined patients’ lymph nodes (*n* = 28; total ROI: 87,696 mm^2^). The highest number of hemophagocytes, this being 370, was found in the hilar lymph nodes (*n* = 28; ROI: 21,924 mm^2^; 370/780; 47.44%; 95%-CI: [43.94, 50.95]). The following other locations were clearly subordinate in terms of the number of hemophagocytes: cervical lymph nodes (206/780; 26.41%; 95%-CI: [23.41, 29.59]), para-aortic lymph nodes (125/780; 16.03%; 95%-CI: [13.58, 18.73]) and inguinal/mesenteric lymph nodes (79/780; 10.13%; 95%-CI: [8.155, 12.4]).

Important data for the 28 patients concerning hemophagocytosis in the lymph nodes are summarized in Table 2.

### 3.3. Hemophagocytosis Is Found in the Bone Marrow of Patients Who Have Died from Severe COVID-19

**Figure 2:**
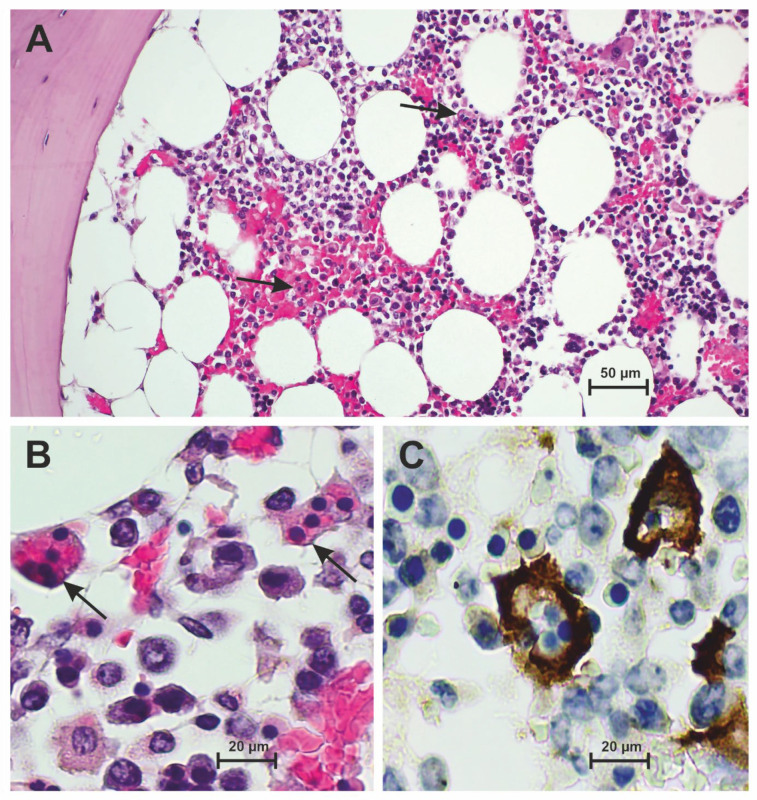
Bone marrow with osseous trabecula in severe COVID-19 disease. (**A**) Partially hemorrhaged bone marrow with architectural disruption and left-shifted hematopoiesis including cells of nucleated and non-nucleated hemophagocytosis (arrows) (H&E). (**B**) Higher magnification of nucleated and non-nucleated hemophagocytosis (arrows) of the bone marrow (H&E). (**C**) Demarcation of hemophagocytes in the anti-CD163 immunostaining.

The histological samples of autopsy-obtained bone marrow showed an intact tissue architecture with lipomatous marrow and trilinear maturing hematopoietic marrow (*n* = 28). In the medullary spaces, there was predominant hypercellularity with repeated accentuation of myeloid cells compared to erythropoiesis (CD71). There was a leftward shift in granulocytopoiesis with a tendency to decrease the number of mature neutrophil granulocytes. The megakaryocytes (CD61) were dissociatively arranged with some formation of microforms and also hyperlobulated cells. An increased number of cellular groups or trabecular association of megakaryocytes could not be distinguished. Dissociative and diffuse arranged T-lymphocytes (CD3), individual B-lymphocytes (CD20), plasma cells (CD138), eosinophilic granulocytes and monocytes (CD163, CD68) were found between the cells of hematopoiesis. Sometimes, there was a slight increase in siderin iron. Histological essentials are illustrated in Figure 2.

A total of 191 nucleated and non-nucleated hemophagocytes were found in all bone marrow samples examined from the patients (*n* = 28; total ROI: 21,924 mm^2^). The severity of hemophagocytosis can be classified on a 3-stage scale [10]. Eight patients (8/28; 28.57%; 95%-CI: [14.24, 47.14]) showed a low degree of hemophagocytosis (1–5 nucleated or non-nucleated hemophagocytes per ROI in the bone marrow). A moderate number of hemophagocytes in the bone marrow (6–10 nucleated or non-nucleated hemophagocytes per ROI) was found in 5 patients (5/28; 17.86%; 95%-CI: [6.851, 35.24]). The number of hemophagocytes in the bone marrow was dramatically increased (> 10 nucleated or non-nucleated hemophagocytes per ROI) in 5 patients (5/28; 17.86%; 95%-CI: [6.851, 35.24]). In the bone marrow of 10 patients, there was no structural evidence of the presence of hemophagocytes (10/28; 35.71%; 95%-CI: [19.77, 54.48]).

Important data for the 28 patients concerning hemophagocytosis in the bone marrow are summarized in Table 2.

### 3.4. Frequency of Hemophagocytosis in Hilar Lymph Nodes Is Higher than in the Bone Marrow of Patients Who Have Died from Severe COVID-19

A total of 780 intranodular hemophagocytes were found in all examined lymph nodes from four different locations (total ROI: 87,696 mm^2^) of the patients (*n* = 28). If the total number of intranodular hemophagocytes (780 in the total ROI: 87,696 mm^2^) was related to the standard area (ROI: 21,924 mm^2^), the average value was 195 hemophagocytes per 21,924 mm^2^ in nodal lymphatic tissue. A total of 191 hemophagocytes were found in all bone marrow samples (ROI: 21,924 mm^2^) examined from the patients (*n* = 28). These values (195 vs. 191) were almost numerically identical.

If the number of hemophagocytes in the bone marrow was compared with that in the lymph nodes derived from an anatomically defined region, a different picture emerges. Based on the standard area (ROI: 21,924 mm^2^), the *t*-test showed a statistically significant difference between the number of hemophagocytes in the bone marrow, 191 (*n* = 28; ROI: 21,924 mm^2^), and in the hilar lymph nodes, 370 (*n* = 28; ROI: 21,924 mm^2^), with *p* < 0.05 (one-tailed: *p* = 0.007; two-tailed: *p* = 0.0146). The MWU test (two-tailed) revealed *p* = 0.003 (Levene 0.045).

The differences in the number of hemophagocytes in the bone marrow (191 hemophagocytes; *n* = 28; ROI: 21,924 mm^2^) and in the examined lymph nodes from the selected anatomically defined locations were not statistically significant using the *t*-test [cervical (206 hemophagocytes; *n* = 28; ROI: 21,924 mm^2^; one-tailed *p*-cervical: 0.381; two-tailed *p*-cervical: 0.7617); para-aortic (125 hemophagocytes; *n* = 28; ROI: 21,924 mm^2^; one-tailed *p*-paraaortic: 0.143; two-tailed *p*-paraaortic: 0.2855); and inguinal/mesenteric (79 hemophagocytes; *n* = 28; ROI: 21,924 mm^2^; one-tailed *p*-inguinal/mesenterial: 0.1; two-tailed *p*-inguinal/mesenterial: 0.1998)]. In addition, the MWU test (two-tailed) was not statistically significant [cervical (206 hemophagocytes; *n* = 28; ROI: 21,924 mm^2^; *p*-cervical: 0.296); para-aortic (125 hemophagocytes; *n* = 28; ROI: 21,924 mm^2^; *p*-para-aortic: 0.958); and inguinal/mesenteric (79 hemophagocytes; *n* = 28; ROI: 21,924 mm^2^; *p*-inguinal/mesenterial: 0.313)].

## 4. Discussion

The data provide evidence that hemophagocytosis in hilar lymphatic nodes (*p* < 0.05), but not in lymphatic nodes from other anatomical sites (*p* > 0.05), is more sensitive than in bone marrow in indicating a severe and lethal course of COVID-19 disease, the infection with severe acute respiratory syndrome coronavirus 2 (SARS-CoV-2).

SARS-CoV-2, a membrane-enclosed RNA virus, is a well-known highly contagious virus and belongs to the genus of betacoronaviruses. Since it is assumed that SARS-CoV-2 only recently passed from the animal reservoir to humans, it is referred to as an “emerging pathogen” that can cause serious illnesses that can be fatal [19]. Typically, in the severe course of the disease, there is pronounced activation and dysregulation of the immune system with significantly increased levels of pro-inflammatory cytokines with inhomogeneous behavior of leukocytes, with an increase in the number of neutrophil granulocytes and a decrease in lymphocytes. The hyperferritinemia observed in SARS-CoV-2 with a pronounced increase in IL-6 is considered as a reliable indicator of uncontrolled activation of macrophages and the manifestation of sHLH [2].

The extraordinary immune activation in the case of sHLH of the bone marrow has already been extensively investigated and proven in severe infectious diseases, especially SARS-CoV-2 [3,20,21]. Typically, susceptibility to sHLH is promoted by blood transfusion and especially by comorbidities including autoimmune diseases, infectious diseases that exist in addition to SARS-CoV-2 and also malignant neoplasms. In the cohort examined (*n* = 28), the majority of patients had comorbidities (25/28; 89.29%; 95%-CI: [73.55, 97.2]), with the majority having more than one additional disease (23/25; 92%; 95%-CI: [76, 98.64]; total 23/28; 82.14%; 95%-CI: [64.76, 93.15]). Three patients also had a malignant tumor disease (3/25; 12%; 95%-CI: [3.145, 29.28]; total 3/28; 10.71%; 95%-CI: [2.799, 26.45]). In this respect, the patient collective showed a constellation of comorbidities that certainly predisposes them to the manifestation of sHLH.

In general, it can be assumed that in septic patients, including in the case of COVID-19, the manifestation of sHLH is associated with a rapid progression of the disease and a poor prognosis [4,22]. Hemophagocytosis in COVID-19 has already been described in numerous works as a morphologically definable phenomenon and has been shown in particular in the bone marrow, indicating a severe course of the disease [23,24,25,26]. In some studies, hemophagocytosis has been found not only in the bone marrow, but also in extramedullary tissues, such as lymph nodes and spleen [27,28]. There was a variable expression of hemophagocytosis in the organ systems with a partial dominance of non-nucleated hemophagocytosis, i.e., erythrophagocytosis.

In a previous work from our group on hemophagocytosis in SARS-CoV-2, the occurrence of hemophagocytosis in the bone marrow was identified as a reliable indicator of severe infection with SARS-CoV-2 with a poor prognosis [10]. In this study and in the work of other groups [29,30,31], locoregional and organ-dependent variabilities in the expression of the phenomenon of hemophagocytosis were not addressed. In particular, there was no comparison of the findings of hemophagocytosis in the predetermined organs such as the bone marrow and lymph nodes. In the current study, the possible existence of locoregional differences in the expression of hemophagocytosis was addressed. For this purpose, lymph nodes from completely different anatomical locations were dissected (hilar, para-aortic, inguinal, mesenteric) and subsequently examined histologically. The morphometric-based evaluation of the frequency of hemophagocytosis in severe COVID-19 disease showed a clear preference for hilar lymph nodes when comparing lymph nodes from the different locations. In addition, a comparison with the bone marrow, which has previously been established as an sHLH indicator in COVID-19 disease, showed a significantly higher sensitivity of the hilar lymph nodes to hemophagocytosis (*p* < 0.05). However, this did not apply to the lymph nodes from topographically different locations.

A possible cause of the observed locoregional differences in the expression of hemophagocytosis in the hilar, para-aortic, inguinal and mesenteric lymph nodes can be their respective topographical proximity with regard to the localization of the inflammatory focus. In relation to COVID-19, diffuse alveolar damage to the lungs is the decisive inflammatory event for the entire organism with pronounced, pro-inflammatory parenchymal destruction. The detection of the virus in other organ systems, such as the heart, liver and kidneys, has been extensively described [32,33], but the associated tissue damage and pro-inflammatory lesions in these tissues are significantly less pronounced than in the lungs. In this respect, the high topographical proximity of the hilar lymph nodes to the lung affected by COVID-19 compared to the lymph nodes of other locations would be seen as relevant for the high susceptibility of the hilar lymph nodes to hemophagocytosis. In addition, the hilar lymph nodes appear as the first draining lymph nodes of the pulmonary system.

In contrast to hemophagocytosis in the bone marrow, which includes both nucleated and non-nucleated (erythocytic) hemophagocytosis, in the lymphoid tissues examined here, hemophagocytosis was clearly dominated by erythrophagocytosis (non-nucleated hemophagocytosis). In the current study, the hilar lymph nodes in the marginal sinus in particular showed pronounced confluent patterns of erythrophagocytes. This finding was not as pronounced in the lymph nodes of the other locations (cervical, para-aortic, inguinal/mesenteric), where the erythrophagocytes were more dissociative. Our data provide further evidence for the concept of compartmentalized human host responses to life-threatening infections such as COVID-19. In contrast to these findings, the HLH-2004 criteria catalog states that hemophagocytosis in the bone marrow, spleen and lymph nodes are to be viewed as equivalent [34].

Limitations of the present study result, among other things, from the partially different medical and paraclinical details of the index group. For example, the pre-existing underlying diseases vary between patients, as well as the number of days with ventilation and the duration of hospitalization. In addition, the number of patients is limited.

Based on our own data, the sensitivity of the biomarker erythrophagocytosis certainly appears to have a threshold value for the presence of sHLH, which was reached and exceeded in the hilar lymph nodes of the examined cohort with COVID-19. The exact determination of the threshold value of erythrophygocytosis is problematic using the available autopsy-obtained study material and without an adequate control group. While the threshold value for erythrophagocytosis of the lymph node has not yet been defined, there is already information to this effect for nucleated hemophagocytosis of the bone marrow [12].

In view of the existing heterogeneity of the host response and the underlying variability, compartment-specific immune reactions, and tissue resilience, a biopsy of hilar or parabronchial lymph nodes in severe COVID-19 disease should be attempted in routine clinical practice. This should be done whenever clinical signs of sHLH become apparent and could be performed at the time of intubation.

## 5. Conclusions

In summary, the current study shows that in severe COVID-19 disease, the hilar lymph nodes show pronounced hemophagocytosis, which indicates sHLH. The extent of hemophagocytosis in the hilar lymph nodes is greater than in the bone marrow. This finding supports the concept of compartmentalized human host responses to life-threatening infection and should be taken into account clinically when selecting biopsy material to confirm sHLH.

## Figures and Tables

**Table 1:** Clinical data for each patient (*n* = 28).

Pat.No.	Age [Years]	Sex	BMI [kg/m^2^]	Hospitalization [Days]	Ventilation/ECMO [Days]	Key Comorbidity
1	82	m	26.1	8	8/0	Diabetes type 2
2	66	m	31.6	6	5/0	Hypertension
3	77	m	25.2	37	8/0	Diabetes type 2
4	54	m	26.6	11	6/2	Chronic lymphatic leukemia
5	80	m	44.2	7	7/0	Diabetes type 2
6	87	w	25.1	13	0/0	Diabetes type 2
7	82	w	26.3	1	0/0	Liver cirrhosis
8	83	w	25.1	7	0/0	Diabetes type 2
9	85	w	23.7	10	0/0	Esophageal squamous carcinoma
10	52	w	25.8	5	0/0	Adenocarcinoma of the colon
11	64	m	34.6	20	16/0	Chronic obstructive lung disease
12	64	m	33.2	15	20/9	-
13	81	m	32.2	31	0/0	Prostatic carcinoma
14	80	m	24.1	4	4/0	Diabetes type 2
15	61	m	28.5	56	16/40	-
16	86	m	26.9	6	0/0	Prostatic carcinoma
17	84	w	21.5	3	0/0	Adenocarcinoma of the rectum
18	80	m	27.1	27	26/0	Hypertension
19	85	w	43.1	9	0/0	Amyloidosis
20	78	m	32.1	16	11/0	Prostatic carcinoma
21	79	m	29.4	22	19/0	Hypertension
22	83	m	25.7	1	0/0	Diabetes type 2
23	66	m	25.7	11	7/0	Liposarcoma
24	83	w	35.2	9	8/0	Hypertension
25	58	m	38.7	9	8/0	Adenocarcinoma of the rectum
26	80	m	36.1	19	16/0	Hypertension
27	62	w	29.7	15	0/0	Hepatocellular carcinoma
28	58	m	32.5	8	0/0	-

**Table 2:** Number of hemophagocytes in bone marrow and lymph nodes for each patient (*n* = 28). N/A: nodal lymphatic tissue not available.

Pat.No.	HLH Score	Number of Hemophagocytes in					
		Bone Marrow	All Lymph Nodes	Hilar Lymph Nodes	Cervical Lymph Nodes	Para-aortic Lymph Nodes	Inguinal/Mesenterial Lymph Nodes
1	146	1	3	3	0	N/A	0
2	191	4	35	13	22	N/A	0
3	127	2	26	2	16	N/A	8
4	160	1	11	7	4	0	0
5	54	0	38	20	7	11	0
6	68	5	6	1	2	3	0
7	47	0	21	0	5	6	10
8	35	38	7	1	1	5	0
9	35	7	9	5	3	0	1
10	54	7	9	4	1	2	2
11	95	0	27	8	3	6	10
12	19	0	11	4	2	1	4
13	42	0	31	25	3	0	3
14	112	9	38	13	10	15	0
15	84	19	68	32	13	3	20
16	91	0	5	2	3	0	0
17	56	0	2	0	0	1	1
18	106	26	71	52	19	0	N/A
19	68	3	25	9	10	0	6
20	106	4	21	3	10	8	0
21	107	35	105	49	33	13	10
22	58	0	17	10	0	5	2
23	35	13	72	35	19	18	N/A
24	91	0	5	0	5	0	0
25	107	7	56	41	3	10	2
26	35	0	11	11	0	0	0
27	126	7	39	17	9	13	N/A
28	58	3	11	3	3	5	0
**Total**		**191**	**780**	**370**	**206**	**125**	**79**

## Data Availability

Additional data are unavailable due to ethical restrictions. Please contact the corresponding author.
